# Spotlight on Assistance Dogs—Legislation, Welfare and Research

**DOI:** 10.3390/ani8080129

**Published:** 2018-07-26

**Authors:** Annika Bremhorst, Paolo Mongillo, Tiffani Howell, Lieta Marinelli

**Affiliations:** 1 Companion Animal Behaviour Group, Division of Animal Welfare, VPHI, University of Bern, 3012 Bern, Switzerland; 2 Animal Behaviour, Cognition and Welfare Research Group, School of Life Sciences, University of Lincoln, Lincoln LN6 7TS, UK; 3 Laboratory of Applied Ethology, Department of Comparative Biomedicine and Food Science, University of Padua, 35020 Legnaro, Italy; paolo.mongillo@unipd.it (P.M.); lieta.marinelli@unipd.it (L.M.); 4 Anthrozoology Research Group, Department of Psychology and Counselling, School of Psychology and Public Health, La Trobe University, Bendigo, VIC 3552, Australia; t.howell@latrobe.edu.au

**Keywords:** dog, assistance dog, guide dog, legislation, welfare, behaviour, cognition, training, genetics, selection

## Abstract

**Simple Summary:**

Assistance dogs support humans with a variety of disabilities. Although guide dogs in particular have a long tradition in Western cultures, the legal situation around assistance dogs has been insufficiently developed in many countries so far—a situation that potentially negatively affects both animal and owner. There is also an insufficient amount of research examining assistance dogs in other areas. Studies investigating assistance dogs’ welfare status, cognitive and behavioural capacities, selection criteria for the best fitting individuals, effective training and management practices, and genetic issues, are so far mainly lacking. This review takes a comprehensive approach—it initially outlines important aspects of the current legal situation for assistance dogs in the European Union and Australia, and then it summarizes research findings focusing on dogs’ welfare, cognition, behaviour, health and training. For each of these areas, the need for future development is highlighted and potential ideas for future directions are discussed.

**Abstract:**

Assistance dogs are a very diverse group of working dogs that are trained to assist humans with different types of disabilities in their daily lives. Despite these dogs’ value for humankind, research on their welfare status, cognitive and behavioural capacities, selection criteria for the best fitting individuals, effective training and management practices, and genetic issues are so far lacking. This review highlights the need to address these topics and to promote progress in legal issues around assistance dogs. The topic of assistance dogs is approached comprehensively by outlining the current status of knowledge in three different dimensions: (1) the legal dimension, outlining important legal issues in the EU and Australia; (2) the welfare dimension; and (3) the dimension of research, covering assistance dog selection and training. For each of these three dimensions, we discuss potential approaches that can be implemented in the future in order to support assistance dog working performance, to protect the dogs’ welfare, and to improve our knowledge about them. Additionally, there remain many legal issues, such as the presence of assistance dogs in public areas, the resolution of which would benefit both the assistance dog and the owner with disability.

## 1. Introduction

Assistance dogs are dogs specifically trained to assist humans with disabilities [1,2]. The term assistance dog itself is an umbrella term, comprising a range of different types of assistance dogs varying in their special training and later purpose (i.e., the tasks they have to perform when living with a person with disability). Although variations in the labelling and definition of the different types of assistance dogs exist, the following types are commonly distinguished: (I) guide dogs; i.e., dogs that assist humans that have visual impairments [2]; (II) hearing dogs; i.e., dogs that assist humans that have hearing impairments [1,2]; (III) service dogs; i.e., dogs that assist humans with mobility impairments [2]. Oftentimes, medical detection dogs trained to alert or respond to medical issues such as human diabetes or epilepsy (with the latter, sometimes further differentiations are made into seizure alert and seizure response dogs, see e.g., Reference [3]) or to assist humans with psychiatric impairments, are classified as service dogs (e.g., Assistance Dogs International, 2009c in: [4]). The definitions of the different types of assistance dogs and the terms used for labelling them are not consistent throughout the literature. Thus, others might define these roles differently. For instance, service dogs can also be defined as being inclusive of both guide dogs and hearing dogs (see e.g., [5]).

Assistance dogs should not be confused with therapy dogs [3]. An important distinguishing factor is that whereas the latter participate in structured, therapeutic interventions with licensed professionals [6] over a restricted amount of time, the former are permanently placed with humans with disabilities to assist in their daily life [4].

Assistance dogs were the topic of a round table panel discussion that was organized as part of the Canine Science Forum, an interdisciplinary conference for experts on canines, held in Padua, Italy, in 2016. A diverse range of invited people participated in this round table, such as scientists, representatives of different government authorities, representatives of different assistance dog organisations, and persons with disabilities. By bringing them together, the round table panel discussion highlighted three essential dimensions relevant to assistance dogs and their owners with disability: (1) the legal dimension; (2) the welfare dimension; and (3) the research dimension.

In this paper, we reflect on important topics that have been brought up by the participants during the discussion of this round table panel and address them in more depth. For each of the three dimensions, both the current status of knowledge in this dimension will be outlined, as well as potential future directions. While the legal dimension addresses the current legal status of assistance dogs in European countries as well as Australia, thereby incorporating both dog—as well as human-related aspects, the welfare dimension and the research dimension both only focus on aspects related to the assistance dog. Studies on assistance dogs have so far primarily focused on the human side, such as the benefits people with disabilities gain from owning an assisting animal (e.g., [7,8]). Our aim, however, is to spotlight the animal side—the assistance dog—addressing aspects relevant to the animal that, in our opinion, should receive more attention in the future.

## 2. The Legal Dimension

### 2.1. Current Status

In Europe, assistance dogs such as guide dogs have been used for several centuries and the first officially recorded assistance dog training schools were founded in Germany after the first World War [9]. Compared to guide dogs, the other types of assistance dogs have evolved rather recently [3]. Nowadays, guide dogs in particular are well established in most European countries, with numbers expected to grow, and an even higher increase in numbers is predicted for the other types of assistance dogs, according to a report that was supported by the European Union Programme PROGRESS ([10]). Despite the long history assistance dogs have in Europe as well as their continuously increasing numbers, we are still lacking comprehensive EU-wide laws dealing with main issues on this special kind of working dogs. However, even on a smaller scale, national laws addressing the different types of assistance dogs comprehensively are mainly insufficient and fragmented, and they differ from each other across the EU countries.

Laws are important, constituting a set of officially recognized rules that provide—in this case people with disabilities—rights which are binding on the whole community and that can be enforced if disobeyed [11]. This lack of comprehensive laws both in the EU, as well as on the national level, has various negative consequences, from more general difficulties concerning the definition and recognition of the different types of assistance dogs (e.g., by government institutions) up to individual challenges in the dogs’ and owners’ everyday life, addressing and securing, for instance, their freedom of movement.

Assistance dogs allow people with disabilities to achieve an improved level of independence [1] and safety. For instance, diabetes alert dogs indicate a potentially fatal blood glucose level, allowing the owner to take the necessary steps to protect him/herself [12]. However, this requires the assistance dogs to have the permission to accompany the human owners in their daily life. Regarding access rights of people with disabilities, a UN convention [13], signed by almost all States Parties, specifically addressed the mobility of persons with disabilities in article 20: “States Parties shall take effective measures to ensure personal mobility […] for persons with disabilities, including by […] forms of live assistance” [13], referring to assistance dogs. However, the implementation of this right still seems to be insufficient, as indicated by a European Parliament document [14] which focused solely on guide dogs in the European Union. This document [14] provides an overview on the access rights of guide dogs in all EU member states, reporting that the legal regulations for even this well-established type of assistance dog differ significantly between the countries. Assistance dog owners still seem to be confronted with difficulties regarding public access and access to means of transportation, as described in a recent UK briefing paper from the House of Commons [15].

Access to means of transportation, in particular, is oftentimes not only important on a national level but even beyond, in order to enable persons with disabilities to travel with their assistance dogs and to cross country borders. Regulations have been implemented by the European Parliament concerning the rights of humans with disabilities when travelling by plane (e.g., [16]). This regulation specifies that recognized assistance dogs should be transported in the cabin; however, it further subjects this to national regulations which can be problematic for assistance dog owners, given the possible discrepancies of national regulations between countries. Furthermore, airlines often have specific regulations for the transportation of assistance dogs. These regulations can differ between airlines, which requires the owner to carefully check each airline’s rules for access permissions of assistance dogs before travelling. Airlines sometimes require the assistance dog to be certified by certain assistance dog provider organisations in order to be allowed to access the cabin. British Airways, for instance, allows assistance dogs to accompany their owner in the airplane if the dog is certified by an organisation that is a full member of Assistance Dogs International (ADI) or of the International Guide Dog Federation (IGDF) [17]. ADI and IGDF are well-known, worldwide organisations for assistance dogs and guide dogs, respectively. However, making the access subject to certification by these organisations could potentially lead to difficulties for handlers accessing the cabin with assistance dogs trained and certified by different organisations. On the contrary, Lufthansa, a German airline, requires the dog to be a “recognized” assistance dog in order to travel with the owner in the cabin free of charge, without limiting this permission to assistance dogs certified from specific organisations [18]. 

While laws and regulations around assistance dogs are lacking on an EU-wide and—for the majority of countries—on a national level, one EU country is outstanding for its progress in this respect. To the authors’ knowledge, so far only Austria has tackled this lack of legal regulations comprehensively, by implementing a nationwide law that deals with the requirements and procedures for the official qualification and recognition of the different types of assistance dogs. This pioneering framework has the potential to be useful for other countries in Europe and beyond. Based on the results of a search for relevant laws conducted using the online database for Austrian laws, https://www.ris.bka.gv.at/, using the search term “Assistenzhunde”, engl. assistance dogs, essential details of this legislation are outlined here.

The Austrian law addressing assistance dogs was amended on 1 January 2015, and integrated into the Austrian Federal Law for Disabled People [Bundesbehindertengesetz]. Article 39a of this law [19] defines the characteristics and requirements to officially qualify and acknowledge a dog as an assistance dog. An additional directive on assistance dogs [Richtlinie Assistenzhunde] [20] further details the practical implementation of this law.

In Austria, different types of dogs assisting humans with disabilities are covered by the umbrella term “assistance dog” [19]:

Guide dogs: Dogs that assist and support blind or severely visually-impaired people;

Signalling dogs: This is itself an umbrella term under which several types of assistance dogs are classified such as those assisting deaf or severely hearing-impaired humans. Dogs assisting humans with chronic diseases such as epilepsy, diabetes or neurological impairments are also classified as signalling dogs;

Service dogs: Dogs that assist humans with physical disabilities and mobility constraints, such as wheelchair users.

Besides specifically defining the different types of assistance dogs, the Austrian legislation dictates the need for an independent coordinating authority responsible for the official examination of candidate assistance dogs. This coordinating authority must be located at an academic institution with expertise in several relevant scientific areas such as veterinary medicine, ethology and animal cognition, as well as ethics and human-animal studies. In Austria, the Messerli Research Institute, which is part of the University of Veterinary Medicine Vienna, was appointed in 2014 to establish the official coordinating authority for assistance dogs in the Republic of Austria.

The directive on assistance dogs [20], referring to the related Austrian law [19], furthermore specifies the requirements and prerequisites that have to be met in order to prove the suitability of a dog to be officially qualified and recognized as an assistance dog:

Health suitability. After a comprehensive veterinary examination, following a standardized protocol that was developed by the independent coordinating authority for assistance dogs, only those dogs that meet the required health standards have the prerequisite to be qualified as an assistance dog.

Behavioural suitability. Only those dogs that show relaxed behaviour (i.e., do not show an intense arousal response) when confronted with diverse environmental stimuli without being significantly distracted in their working performance have the prerequisite to be qualified as an assistance dog. Furthermore, a high obedience level is required. Both aspects are assessed together with the dog’s working performance.

Working performance suitability. The Austrian legislation furthermore states that for qualification as an assistance dog the working performance of the dog has to be officially examined by the independent coordinating authority for assistance dogs. The official assessment of the working performance of the candidate assistance dog is executed in a two-step procedure. In the first assessment, called the “quality assessment”, the dog is evaluated together with the dog trainer by a committee evaluating the dog’s behaviour and obedience (see ‘behavioural suitability’), as well as the working performance required for this specific type of assistance dog. The examination committee always consists of an expert in dog behaviour and training, a person with disability/ies that is experienced with the type of assistance dog to be assessed, and an examination chair. Only after having successfully passed this first assessment, is the dog trainer allowed to permanently hand the dog over to the subsequent new owner (i.e., a person with the type of disability for which the dog was trained to assist).

After the assistance dog has lived and worked together with the new owner for a reasonable amount of time, the second official assessment, called the “team assessment”, is executed by the coordinating authority. Only after this second assessment is passed successfully as well, is the dog officially qualified and recognized as an assistance dog by the Republic of Austria.

This qualification enables the owner to apply for financial support for his or her assistance dog from public funds as well as to officially register the dog as an assistance dog on their ID card, which confirms their status as having a disability. Although access permissions for assistance dogs are not specifically defined by the Austrian Federal Law on Disability, the respective directive [20] specifies the requirement of assistance dogs to enter public places and buildings, as well as the exclusion from the duty to be leashed and muzzled in places and situations where this is required for pet dogs (e.g., on public transport, pet dogs need to be muzzled and leashed in Austria, which is not required for assistance dogs [21]).

The procedure implemented in the Austrian legislation can be considered to protect the security of all involved parties: The dog trainer in terms of seller protection, and the human with disabilities in terms of consumer protection; both the health and the quality of the dog’s working performance are officially assessed before the dog is handed over to the new permanent owner. Furthermore, and not least, it intends to protect the dog specifically by assessing its suitability with regard to health, behaviour, and training status, helping ensure the dog’s welfare.

Looking beyond the European border, there are other countries that have already implemented laws addressing the definition and function of assistance dogs, such as Australia. Having a closer look at the Australian system can provide further beneficial insights into potential frameworks, approaches, and caveats that must be recognized.

In Australia, the law surrounding the use of assistance dogs is clear, at least in principle. A search of the Australian Government Federal Register of Legislation (https://www.legislation.gov.au/) was conducted using the following search terms: assistance animal, animal, assistance, disability, service animal, service. One law in particular is relevant for defining assistance dogs. According to the Disability Discrimination Act 1999/2009, Section 9 (provided here verbatim), an assistance animal is a dog or another animal that is:

(a) accredited under a law of a State or Territory that provides for the accreditation of animals trained to assist a person with a disability to alleviate the effect of the disability; or
(b) accredited by an animal training organisation prescribed by the regulations for the purposes of this paragraph; or
(c) trained:
  (i) to assist a person with a disability to alleviate the effect of the disability; and
  (ii) to meet standards of hygiene and behaviour that are appropriate for an animal in a public place.

In principle, this means that any animal that meets the training requirements of behaviour and hygiene for public access, and helps mitigate the effects of an owner’s disability, is an assistance animal. In practice, the reality is far less clear. The main limitation of enforcing the law as written is that there is currently no official state, territory, or federal accreditation system for assistance animals, although the state of Queensland does require assistance dog providers and trainers to be registered with the state as part of the dog’s qualification [22]. Governments at all levels rely primarily on the assistance animal provider organisations to accredit their own animals for assistance work. Fortunately, according to a recently completed review for the Australian government, providers currently working in this area take their roles very seriously, and are committed to improving outcomes for individuals with disability [23]. Therefore, there currently appear to be no attempts in Australia to take advantage of the accreditation gap by accrediting dogs not well-suited to service roles, in order to make money for themselves. There are no guarantees that this will remain the case in the long-term. Indeed, a large piece of legislation that was passed in 2013 will increase funding for disability supports within Australia. As of March 2018, AUD$15b in funding has been committed to a new scheme that is currently rolling out nationwide. It provides disability support for approximately 160,000 people, 44,000 of whom have never accessed disability supports previously [24]. This potential boost in funding could encourage unscrupulous providers to enter the assistance dog sector.

The National Disability Insurance Scheme (NDIS) Act 2013 provides a national basis for meeting the extra costs to participate in society that individuals younger than 65 years incur because of their disability. It is not intended to replace accessible environments, the responsibility of mainstream organisations (e.g., health) to avoid discrimination against people with disability, or the informal support that people in society rely on. A key feature of the NDIS is that each individual has a tailored plan that reflects goals, outcomes and the supports they will need. A main requirement of the NDIS Act is that the list of supports that are funded by the government must be considered “reasonable and necessary”. With that in mind, the agency responsible for NDIS management contracted a research team in Victoria, Australia, to review existing evidence around whether assistance animals would represent an effective support to address the NDIS participant’s needs. Based on the results of the review [23], the NDIS has agreed to provide assistance animals, but on a case-by-case basis. Given the large size of the NDIS, it is possible that provider organisations could be established simply to gain access to some of this funding, without considering the needs of the individual with disability. While there is no current evidence of this for assistance dogs, there have been anecdotal reports of other disability sectors taking advantage of this lack of regulation (e.g., day care providers for people with disability; [25]). To avoid this possibility, the authors of the review [23] suggested to develop a nationally recognised, independent accreditation scheme for any assistance animal provided as part of the NDIS.

The Australian situation indicates that, even if laws covering assistance dogs exist, implementing these laws into practice is far less straightforward. An independent assessment of each assistance dog to be officially qualified and recognized, instead of being assessed only by the training facility, would be important to ensure objectivity, providing an important way to progress in the future.

### 2.2. Future Directions in Assistance Dog Legislation

Several legal aspects around assistance dogs should be addressed in the future. Currently, we lack comprehensive national laws in many countries as well as EU-wide laws comprehensively addressing assistance dog considerations. Potential future laws at both levels could aim to cover fundamental aspects such as a common definition of the different types of assistance dogs as well as the implementation of an official qualification procedure for assistance dogs (i.e., an independent and official state, territory, federal or EU-wide qualification and recognition procedure for assistance dogs assessing the dogs’ suitability for their specific tasks, their health and behaviour as well as their working performance). Finally, the clarification of rights covering qualified assistance dogs when they are accompanying their owners would be helpful. With the latter, access permissions of assistance dogs in public areas, as well as in means of transportation, are an important topic to be addressed comprehensively in the future in order to improve the dogs’ and owners’ lives.

Addressing issues on assistance dogs on a broader European level is the aim of a standardisation programme launched in 2017 by the European Committee for Standardization (CEN). The objectives of this standardisation programme [26] are, among other things, to develop EU-wide standards with regard to the terminology and definition of assistance dogs, as well as for their training and assessment. This European standardisation programme should “improve visibility and liability of such dogs and their users and improve their lawful right of access to public transport or built environment” ([26], p. 4). By replacing standards at the national level, recognized standards across Europe should be developed by this programme. However, in this context, it is important to point out that standardisations are not binding for member countries, as they are not laws. Nevertheless, standardisation programmes are an important first step, as they can set the direction of future laws implemented in the area.

A further topic to address in the future is the creation of official registration systems for assistance dogs (see Reference [15]). These registration systems could provide government institutions with an overview of all assistance dogs officially qualified and recognized in terms of numbers, owner IDs, and working performance assessment outcomes. However, depending on the individual country’s legislations, privacy regulations (i.e., the use of personal information) might differ, influencing the implementation and feasibility of such registration systems. With the comprehensive inclusion of assistance dogs in their national laws, Austria has also implemented such an official registration system and database of all assistance dogs officially qualified in the country. This database provides improved transparency for government institutions with regard to dog and human data (number of assistance dogs, distribution, age, breed, assessment results, etc.), but it also provides the possibility to track assistance dogs, to assess their welfare state in case concerns might arise, and to ask assistance dog owners to participate in dog training workshops in order to improve their individual knowledge and training skills around dogs in general and assistance dogs in particular. After all, ongoing maintenance training for both the dog and owner may be an important component of a successful assistance dog and handler partnership. On the basis of this database, assistance dogs in Austria even receive an individualised ID emergency card which is attached to the dog’s harness. On this card, a QR-code (two-dimensional barcode that contains information readable by a smartphone with an appropriate app) provides data about relevant people to contact in case there is either an emergency with the assistance dog owner or with the dog. Therefore, Austria has introduced an important security step for both the human and the dog, representing a pioneering example for future progresses in other European countries and beyond.

## 3. The Dimension of Welfare

### 3.1. Current Status

Animal welfare must be a primary consideration in working dogs of any kind, as public opinion in many parts of the world moves toward a greater degree of concern for animal welfare [27]. Despite this, after searching Google Scholar and Web of Science using the terms: assistance dog welfare, assistance animal welfare, working dog welfare, and service dog welfare, we could find only five peer-reviewed scientific journal publications examining assistance dog welfare [28,29,30,31,32]. A book chapter also highlighted potential welfare problems for animals working in assistance and therapy contexts [33]. Why this is the case is not immediately clear; in general, it may be assumed that pet animals living in human homes may experience more positive welfare than, say, livestock animals, but this assumption is not always accurate [34], and assistance dogs are not pets. There is somewhat more research examining welfare outcomes in working dogs in general or in other contexts, such as the military and farming; for a review, see [35]. Some of those findings and recommendations may also apply to assistance dogs, especially issues relating to the effects of long-term kennelling, since many provider organisations kennel their dogs for part of the training.

One of the five studies focused on assistance dog welfare asked owners a series of questions about the quality of their relationship with their dog [29]. The authors interpreted the high value that owners placed on the dog-owner relationship as evidence that the owners prioritise the dog’s welfare and that the dogs, therefore, are experiencing a positive welfare state [29]. A connection between relationship quality and animal welfare seems intuitive, but there is no existing evidence, to our knowledge, to confirm that the link does in fact exist.

Another study also used owner reports to measure the perceived quality of life of pet dogs living in homes, rather than assistance dogs [32]. Nonetheless, it is relevant for this discussion because half of the participants interviewed were parents of children with a developmental disorder, while the other half were parents of neuro-typical children. According to this study, which collected qualitative data through interviews, the benefits of living with children, neuro-typical or otherwise, for dogs were having a daily routine and plenty of play activities. Potential problems included rough play or handling by the child and over-stimulation when the child’s friends came to visit; and this was true for both groups. The stress indicators noted by parents of neuro-typical children varied somewhat from parents of children with a developmental disorder. Parents of the latter children were more likely to mention that the whites of the dog’s eyes were visible, while parents of neuro-typical children mentioned running away from the situation. The authors believe that the reason for this difference, especially in noting the visibility of the sclera among parents of children with a developmental disorder, may be due to many of these parents having previously attended assistance dog training seminars, in which they learned about stress responses in dogs [32].

The other three studies highlight specific concerns for assistance dogs. One of these used qualitative interview data with parent owners of autism assistance dogs for their children, as well as casual observations, to note welfare concerns for this type of dog [30]. Issues such as lack of a daily routine, lack of sufficient ‘time off’, being overweight, and unintentional harm by the child with autism spectrum disorder were highlighted. While rough handling was also noted by pet owners with children [32], the current study expressed concern about the lack of a daily routine, while Hall and collaborators [32] indicated that the pet dogs in their study benefited from having a routine.

The remaining two studies took an ergonomic approach to understanding the difficulties that assistance dogs can face in their work. The first study focused on the challenges that mobility assistance dogs regularly encounter in pulling their owner in a wheelchair, and in opening doors [28]. In direct observations of assistance dogs and their owners, this study found that the angles at which dogs are required to pull the owner and open doors are not efficient for dog or owner, requiring more force to accomplish the goal than would be necessary at other angles. Furthermore, some dogs were overweight, and sometimes the harness did not fit correctly. All of these factors could negatively affect the dog’s health [28], and a harness that does not fit correctly could cause pain at the time of movement, by digging into the animal’s shoulders [28]. A more recent study followed on from this work and found that the type of harness used by guide dog organisations can make a difference in the amount and distribution of pressure placed on the dog [31]. For instance, there was virtually always some pressure being exerted on the right and left sides of the sternum for all harnesses, but there was less pressure when dogs wore a harness with a rigid attachment to the frame with a padded back, compared to harnesses which relied primarily on loops to restrict movement. Fortunately, little pressure was exerted on the dog’s back with either harness, and because each dog-handler team will vary slightly in their movements, the authors did not make any specific recommendations for a preferred harness overall [31].

These studies indicate that there can be clear negative welfare outcomes for at least some assistance dogs. More research of this kind is needed to determine the extent to which these problems exist, as small samples were used in both studies. Assistance dog provider organisations should use these studies as cautionary tales, and work to ensure that their procedures adequately address these potential concerns.

The priority placed on assistance dog welfare is evident in discussions with provider organisations and owners. In an attempt to understand provider organisation policies and procedures for animal welfare, researchers in Australia surveyed providers worldwide [23]. When asked to describe how they ensure dog welfare using a free-text response, nearly all participants indicated that the dog was regularly checked by a veterinarian. Several respondents also mentioned other aspects of welfare, such as an acknowledgement that too much stress was detrimental to welfare, the importance of a proper diet, and the use of only reward-based training techniques. That these comments occurred less frequently than comments about physical health is somewhat concerning. It may be that some provider organisation representatives are not considering all aspects of dog welfare, but it could also be that the intentionally vague wording of the question, “How do you ensure the welfare of your animals during training, working life and beyond?” [23], led participants to focus primarily on physical health. If respondents had been asked about specific factors affecting welfare, the results may have reflected a more holistic perspective. However, in the round table on assistance dogs at the Canine Science Forum in July 2016, there was also a heavy focus on welfare in terms of physical health. One reason for this may be that physical health is one area in which all stakeholders can agree.

During the round table, there was a discussion around the need to develop breeding programmes for assistance dogs. One participant indicated that programme development requires cooperation among multiple stakeholders, such as assistance dog provider organisations, people with disability, and others. A prerequisite is the definition of breeding goals, but these may vary by stakeholder, because the tasks demanded of assistance dogs are very diverse. This can make it difficult to come to one conclusive solution. Furthermore, there was some argument that breeding programmes may not even be desirable, because plenty already exist, and because they necessarily limit the gene pool, a practice which is responsible for a large number of health problems in dog breeds [36]. In theory, it is possible to source shelter dogs to help get around this problem [5]. However, it is impossible to control for the dog’s background when adopting shelter dogs, and whether this impacts on training success rates is unclear. Despite the challenges inherent in developing breeding programmes and other alternatives for dog selection, and the arguments for and against developing one in the first place, a starting point that all stakeholders would agree on, was that the assistance dog must be healthy. It was therefore suggested that specific plans could be developed to select against the presence of genetic diseases.

While providers focused primarily, but not exclusively, on physical health of their dogs, owners may view welfare more holistically. Assistance dog owners were interviewed as part of the Australian study [23]; during the interviews, owners were asked to discuss how they met their dog’s welfare needs. Participants acknowledged the importance of downtime and indicated that their dog had plenty of opportunities for free play. Owners of assistance dogs for children with a developmental disability noted the possibility of the child mishandling the dog and took steps to ensure that the dog was always able to choose whether to remain in the company of the child. Since these were two of the main concerns highlighted in a study conducted by Burrows and colleagues [30], the awareness of these potential problems by owners is encouraging.

### 3.2. Future Directions in Assistance Dog Welfare

Very little research has been undertaken to understand the specific challenges faced by assistance dogs which could negatively impact their welfare, and this needs to change. There is increased variation in the roles in which dogs are employed within assistance contexts, from guiding the visually-impaired to mobility assistance, autism assistance, psychiatric assistance, and alerting when an owner is about to have an epileptic seizure or a low blood sugar episode [23]. We therefore advocate large-scale, long-term studies using objective and multiple measures of welfare, in order to characterise the most pressing concerns for assistance dogs and to determine where progress is being made over time.

In particular, we could find no research focusing on the effects of different training styles on assistance dog welfare, and nothing about how to handle the transition to retirement. There is evidence that using punishment in training for pet dogs is associated with undesirable behaviours and potentially negative welfare outcomes [37], but it is unclear how commonly punishment is used by assistance dog trainers or whether that has long-term negative impacts on this particular population. Furthermore, best practice in transitioning the dog to retirement is currently unknown. Remaining with the owner would likely be the least disruptive to the dog’s life, but the dog would still need to adjust to not going everywhere with the owner. Recommendations for optimising welfare working dogs are available [38], but more research is needed to expand the evidence base and give more specific recommendations for managing retirement. Because all research is contingent on sufficient funding and most provider organisations are non-profit or charity organisations, we call on governments to prioritize funding for research aimed at improving assistance dog welfare.

In the meantime, provider organisations should aim to ensure that they operationalise dog welfare in a way that moves beyond physical health and incorporates psychological well-being. This appears to already be the case among many organisations; it may be true of all organisations, but the current lack of information available makes it impossible to know for sure. Providers should also take care to monitor the dogs’ welfare once it is placed with an owner, and owners should be informed about the practices that they must undertake to ensure animal welfare. Again, this is likely already a common practice among providers, but efforts should be made to confirm this.

Loss of interest in performing the working role can be an indicator of a negative welfare state in assistance dogs [30], so an assistance dog that experiences positive welfare should be a more enthusiastic and effective aid to their owner with a disability. Ensuring good welfare outcomes will therefore improve quality of life for both parties. Loss of motivation to work may be misinterpreted by owners in some cases, so it is important for the owners to receive continuing education about dog behaviour and assistance dog management. This should be provided by the assistance dog provider as part of their ongoing support; veterinarians should also keep this in mind if the owner expresses concern about the dog’s behaviour during routine visits.

## 4. The Dimension of Research

### 4.1. Current Status

For this dimension, a search for articles was performed on Scopus^®^ (https://www.scopus.com/) with the keywords ‘guide dog’, ‘service dog’ and ‘assistance dog’. In general terms, the scientific production on this topic has been rather limited. Only full papers published in peer-reviewed journals, and reporting original research focusing on the dog as the object of the study (and not, for instance, its effect on humans, or the behaviour or traits of their owners or users), were considered. A total of 59 papers were retrieved by this search, of which 52 were actually used for the review. The remaining seven papers were not used due to difficulty in obtaining the full-text, or for the marginal involvement of assistance dogs in the actual research. Most of these studies were published after 1995 (four papers were published between 1982 and 1986 by a single group, and one was dated 1959). With seven studies published, 2017 was the year with the highest number of papers on the topic.

Research on assistance dogs can be split into two broad categories: Those that have a direct applied value for the assistance dog population and those that take advantage of the peculiar characteristics of that population to address scientific questions that are of interest for other dog populations (e.g., working dogs in general) or for the species at large. Several studies tap into aspects that would fit into both categories, but in most cases the declared aims could be used to attribute studies to one category without ambiguity.

In the present paper, more ample coverage will be given to the first category, which can be further classified into four subject areas: the development of performance assessment tools, with predictive value on the individual dog’s success as an assistance dog, which could be used as a basis for selection (Section 4.1.1); the effectiveness of training and management practices in improving the success of assistance dogs (Section 4.1.2); the genetics of relevant traits, which could be used as a basis for selective breeding (Section 4.1.3); and descriptive behavioural studies (Section 4.1.4).

#### 4.1.1. Predictive Value of Tools for Early Selection of Assistance Dogs

Training assistance dogs is highly consuming in terms of time and financial resources, and a sizeable proportion of dogs entering training are withdrawn before its conclusion [39,40], mainly for behavioural reasons [41]. Therefore, a relatively large number of studies in the field aimed at developing tools for the assessment of traits, which could reliably predict success in training. The most frequent approach employs behavioural tests, specifically designed to tap into different dimensions of a dogs’ personality/temperament, which may be relevant to their trainability and future suitability as an assistance dog. Being performed in controlled conditions, these methods provide relatively objective measures of dogs’ reactions to standardized sets of stimuli/situations, although this may also be considered a limitation. In several studies, such tests were utilized as a stand-alone assessment [42,43,44,45], while in others the assessment was complemented by other evaluations, such as behaviours expressed in non-standardized conditions [46], measures of behavioural lateralization [47], physiological parameters [46,47], or environmental factors, such as maternal styles [48].

Another common approach to the assessment of predictive behavioural traits is through questionnaires, investigating specific dimensions (distractibility [49], or personality at large [41,50,51,52,53]), mainly with the aim of creating scales that could guide the early selection of dogs to be sent to training. Questionnaires are a relatively quick and inexpensive way of collecting information about the dogs’ responses to a variety of naturally occurring situations. However, one disadvantage of questionnaires is the inter-individual variability of respondents, as well as different forms of biases (e.g., recall bias, selection bias; for an overview of possible biases in questionnaires, see [54]) and the fact that many users are not sufficiently knowledgeable to assess behaviour/welfare [55]. These limitations require the tool to undergo a laborious validation, for it to provide usable data. The extent and completeness of validation varies between the studies, but only Harvey and colleagues [52] provided a comprehensive account of the development and validation process. Duffy and Serpell’s study [51] was the only study relying on a pre-existing, previously validated [56] personality assessment, the C-BARQ. Finally, three studies assessed traits unrelated to temperament or behaviour as their main predictors, namely salivary immunoglobulin A [57], various measures of lateralization [58] and functional magnetic resonance responses to the presentation of trained signals [59].

In all the aforementioned studies, some degree of correlation was found between at least one of the measures/traits and training outcomes. However, it was surprising to find that few studies analysed and reported measures of the assessments’ suitability as predictive tools. Among those that do so, Batt and collaborators study [47] is the only one reporting an overall accuracy of 100% for one of their measures (assessment at 14 months), in predicting positive and negative outcomes; however, the small sample size (*N* = 43) imposes caution in the generalizability of these results. The overall accuracy of other behavioural assessment tools falls between 64% and 87% [41,43,45]; however, a large variability exists in terms of sensitivity in detecting failures (range: 3–85%), positive predictive value (i.e., the probability that a dog indicated as likely unsuccessful by the assessment, will actually be withdrawn from the program; range: 8.4–93.3%), and negative predictive value (i.e., the probability that a dog indicated as unlikely to be unsuccessful by the assessment, will not be withdrawn from the program; range: 8.4–93.3%). By contrast, specificity in detecting failures was less variable and generally good (range: 81.8–99.6%). In other words, the chief advantage of all these assessments is that they rarely attribute a withdrawal judgment to dogs that should not be withdrawn. In addition, improvement in predictive values are reported when physiological predictors are used in combination with behavioural evaluations [57,59], suggesting that a multi-dimensional approach may be more valuable than tools looking into single aspects. Curiously, none of the examined studies combined the two most common methods of assessment, i.e., questionnaires and behavioural tests.

The cost of producing assistance dogs can be reduced by rehoming non-suitable dogs at an earlier stage and allowing more resources to be used on dogs with a greater potential. Therefore, predictive measures should be provided as early as possible. Unfortunately, an early assessment may lose predictive power, as the developing dog’s behaviour is deemed to change over the first months of life [60]. Thus, one challenge is to find the age point that increases the benefit/cost ratio. Some assessments are performed as early as at 6–8 weeks of age [45], others in the early adulthood (15–20 months [42]), but most are performed during the juvenile period, while dogs are fostered to puppy walkers, or just before entering training. Comparing the same assessment performed at different ages would provide important information regarding the stability of the trait and the most appropriate time for the assessment. However, only a handful of studies performed repeated assessments [43,47,51,52] and the resulting picture is rather varied. For instance, Batt and collaborators [47] indicated 14 months as the age at which their assessment gives the best predictive value, while others [43] have reported similar predictive values for 5 or 8 months, suggesting that the ideal age depends on the type of assessment. In addition, the nature of the prediction may also influence the ideal age at which assessment should be performed. For instance, Harvey and collaborators [52] reported their 5 month assessment to be only reliable in predicting a positive, but not a negative, outcome of training, whereas the 8 and 12 month assessments were able to predict both positive and negative outcomes.

An additional aim shared by most of these studies is to identify behavioural traits that, regardless of their predictive value, are associated with later success in training. In turn, such traits could represent a specific aspect to be investigated in other studies, or a target of potential interventions aimed at improving training success. As already mentioned, all of the studies identified some correlation between at least one of their measures and training success. However, it is difficult to provide a comprehensive picture, as important differences in how individual behaviours were defined, measured and interpreted in different studies hinder the possibility to compare and generalize for most of the traits. In general terms, two personality dimensions were recursively suggested to being negatively related to success, namely anxiety [43,46,48,52], and distractibility [41,43,52], which may therefore represent the two most important traits to this regard.

#### 4.1.2. Effectiveness of Assistance Dog Training and Management Practices

Very few studies looked at the overall effectiveness of management or training practices in improving the success of assistance dogs, unexpectedly making this a scarcely explored domain, given its potential implications. Batt and colleagues [61] investigated the effect of a number of management practices in place at various organisations at the international level, on training and working success rate (the latter being the rate of dogs still working as guide dog one year after completing training, a measure the authors considered more important than merely completing the training program) of dogs entering their training programs. The investigated factors included breeds employed; breeding policies; the source of dogs; the use of puppy raisers; the ways in which feedback from puppy raisers was obtained; the use, timing and type of temperament tests; and the application of quotas. Factors associated with working success included the use of tests for the selection of puppies and that of in-field assessments, but not of structured temperament tests for adult dogs; working success was also associated with receiving feedback from puppy raisers through questionnaires, but not through interviews, and from staff when making decisions about the puppies. Using external breeders and using Labrador/Golden retriever crosses was associated with greater working success, as was the use of quotas (i.e., setting the number of graduating dogs in accordance with the demand). Although the study was not designed to determine the causal nature of the results, it does highlight some important features of management which warrant further investigation.

Two studies looked more specifically at the effectiveness, in terms of improving success rates, of training-related practices. The pilot study by Batt and colleagues [47] reports no effect of a 5-week program of either training or socialization on puppies’ later success as guide dogs, although methodological limitations (e.g., small sample sizes and existence of subpopulations within groups, as acknowledged by the authors) restrict the breadth of the conclusions. Ennik and co-authors [39] published a retrospective study looking at factors influencing the rate of success in a large sample (*N* = 2033) of potential guide dogs. The study shows that dogs being passed back to an earlier stage of training for behavioural reasons had lower chances of becoming guide dogs, but being passed back for other reasons (i.e., medical reasons that delayed the completion of training, or lack of a suitable owner match in the current class) was beneficial in terms of final success rate, though only for certain breeds. Finally, one study looked at the efficacy of behaviours used by puppy walkers to cope with inappropriate behaviours expressed by candidate guide dog puppies. Rejecting or not responding to the dog were effective ways of dealing with biting, while physical control was more effective in preventing damage to objects [62]. Overall, there is very limited information regarding optimal training or management practices, making this an area of potential great improvement.

#### 4.1.3. Genetics of Relevant Traits for Selective Breeding of Assistance Dogs

The improvement of a population’s genetics for relevant traits is a founding element of animal breeding. For assistance dogs, the small diversity of breeds employed, the fact that many organisations control their own breeding stocks and that records are often kept of their dogs’ performance, make selective breeding a promising source of improvement in the field. Selection programs can only be successfully implemented for traits with a sufficient degree of heritability, a parameter that expresses the proportion of the phenotypic variance that is attributable to additive genetic variation. There are few data about the heritability of traits of interest for assistance dogs. Most were produced by a single research group more than 30 years ago [63,64,65], while only one study was carried out in recent years [66]. The latter provided heritability estimates of dogs’ responses to a standardized and validated behavioural test, administered to 6-week old Labrador retrievers, Golden retrievers, and crosses between the two breeds. Only moderate heritability was found, and only for four of the 11 investigated traits (i.e., following, retrieve-response to stimulus, retrieve-response to assessor and tunnel). Although, theoretically, selection of breeding stocks could be based on such traits, these were not predictive of success in training as a guide dog [45], raising doubts about the usefulness of such genetic selection. By contrast, Goddard and Beilharz [63] used dogs’ training records to estimate the heritability of the success in becoming a guide dog or of the reasons for failure, which resulted in relatively high heritability (0.44). Fearfulness, which the study identified as the most important and heritable component of failure, was later investigated in greater detail [65], showing that genetic factors explain most of the variability in general fearfulness, rather than fear of specific stimuli, suggesting the former as the main trait on which to base selection of breeding stocks.

#### 4.1.4. Descriptive Behavioural Studies

This category includes studies that mainly produced descriptive results. Nonetheless, these studies have a potentially applied value, as they depict relevant situations specific to assistance dogs that could guide choices at the practical level, in addition to providing directions for future investigations. Gazzano and co-authors [67] asked puppy walkers of prospective guide dogs to report on undesirable behaviours expressed by their puppies; about 65% of dogs expressed at least one such behaviour, but only a relatively small percentage of these is represented by behaviours of particular concern for their future enrolment as guide dogs (e.g., aggression or social fear). Two studies provide a description of interactions between prospective assistance dogs and their carers/trainers [68,69]. An interesting finding of Koda [69] is that the quality of play changed over time, with progressively less time spent fighting and chasing and more time spent in more gentle and controlled types of play (e.g., holding a toy together without tugging), pointing out the potential role of the puppy walker in curbing undesirable behaviours.

As mentioned before, a last category includes studies that—while involving assistance dogs as subjects—do not primarily aim at providing results with an applied value for that field. In several cases, the rationale for employing assistance dogs was to take advantage of some traits peculiar to that population, which represent ideal conditions to investigate biological phenomena of relevance for dogs at large. For instance, the specific training and subsequent intense relationship with a person with a visual impairment provide the grounds to examine the role of experience on the development of dogs’ interspecific socio-cognitive behaviour, including dogs’ ability to respond to humans’ referential communication [70] or to their attentional states [71], or dogs’ behaviour when asking humans for food [72], for toys/playing [73], or for support when facing a task they cannot solve on their own [74]. Notably, all of these studies failed to find differences between pets and working guide dogs, likely because the latter do experience regular interactions with sighted people even after they are assigned to a person with a visual impairment. In fact, only trained but not yet working guide dogs were found to differ from both pets and working dogs, with the former looking less at humans when facing an impossible task, highlighting the role of training on human-directed gazing [74]. The effect of experience on the expression of dog-human attachment behaviour is another aspect that was investigated in guide dogs, on the premise that their training regime typically involves the formation and breakdown of two strong relationships—first with the puppy walker and then with the trainer, before eventually being able to develop an enduring relationship with their assigned handler. In spite of such recurring relational disruptions, dogs still show a full-fledged attachment behaviour towards their handler [75]; however, experience does impact on how such behaviour is expressed, since, compared to pet dogs, guide dogs show greater behavioural control [76], but higher cardiac activation [77], when separated from their owner. One study looked at the effects of guide dog training on dog-human cooperative behaviour, suggesting the existence of differences as well as similarities with pet dogs [78], although the descriptive nature of the study limits the scope of the results.

In some cases, the guide dogs’ population was used as a model for investigating aspects of relevance for working dogs in general, such as health issues in aged working dogs [79,80]. In many other cases, the involvement of guide dogs is justified by more generic advantages of the population, such as being relatively homogeneous for some characteristics (e.g., in terms of breeds, early life experience, presence of track records of performance). These studies are more varied in scope, spanning cognition [81], behavioural lateralization [82,83], temperament [81,83,84,85], behavioural development [86,87], behavioural genetics [88], or reproductive physiology and behaviour [89,90,91].

### 4.2. Future Directions in Assistance Dog Research

The review of the existing literature highlighted some critical points in scientific research on assistance dogs. The assistance dog sub-population involved in these studies is generally guide dogs. However similar, the specificities of guide dogs may not be the same as those of other types of assistance dogs, such as those assisting people with hearing or mobility impairments. Although guide dogs may represent the largest subset of assistance dogs, future studies should expand their scope to include other categories.

There have been some studies looking at reliable predictors of dog training success. Methodological considerations, including validation processes, completeness of data, and the definition of traits of interest, are extremely variable, hindering the reliability of the results and the possibility to provide a comprehensive evaluation of such studies. Future studies should strive to increase methodological soundness and possibly to achieve some uniformity in terminology and definitions. Overall, research indicates that no single assessment tool exists that possesses ideal predictive characteristics. Some studies suggest that combined assessments provide better predictive values than those employing a single method. However, no study seems to have evaluated the combination of the most common and feasible type of assessment (i.e., questionnaire) with other types of assessment, such as behavioural tests and/or physiological measures, which represent a promising avenue for future implementation of predictive tools.

One aspect that unexpectedly received scarce attention is the effectiveness of training and management practices. Scientific evaluation is likely to be hindered by practical issues. For instance, there may be resistance by the organisations, which would have to invest time and resources in management/training practices with unknown effectiveness, with the risk of increasing the number of dogs that do not reach acceptable standards. The same risk does not exist in studies looking at the suitability of assessment methods, which can be performed in addition to, rather than replacing, existing ones. However, it is easy to understand that the potential long-term benefit of these studies is great, as an optimization of management and training practices would certainly allow organisations to use their limited resources more effectively, achieving better performance in the long-term.

One critical issue of current research is that the vast majority of the studies were carried out using populations from one specific assistance dog organisation. However, relevant differences exist in the procedures in use by different associations, even within the same country, which may make the results of the study only valid for the specific population of dogs in which the study was conducted. In fact, the only study that specifically explored this possibility, reports that the predictive value of the method varied significantly depending on which organisation the tested dogs belonged to [51]. Therefore, while it is difficult to achieve uniformity across associations and countries in management, training, and evaluation practices, more studies should scientifically investigate differences between practices and to what extent the results of studies can be generalized to the assistance dog population, regardless of their source.

## 5. Conclusions

Our aim here was to spotlight assistance dogs and to elaborate on the legal, welfare, and research dimensions around these special kinds of working dogs, as discussed at the Canine Science Forum in 2016; all three covered the current status as well as provided ideas about prospective future steps. As pointed out, in each of these dimensions, different achievements have so far been realized—be it in terms of implementing assistance dogs in legislations to guarantee certain permissions to them and rights for their owners, or in conducting research to gain new insights into the dogs’ welfare state, behaviour, and cognitive capabilities, as well as in terms of breeding, individual selection and efficient training. However, simultaneously, we still face significant knowledge gaps and important improvements to be addressed in the future. We encourage putting assistance dogs into stronger focus, not only in legislation but also in research to increase our knowledge and awareness about them. This will benefit the dogs themselves, and will promote further appreciation of the significant role assistance dogs play for people with disabilities.
